# Unfolding of Helical Poly(*L*-Glutamic Acid) in *N*,*N*-Dimethylformamide Probed by Pyrene Excimer Fluorescence (PEF)

**DOI:** 10.3390/polym13111690

**Published:** 2021-05-22

**Authors:** Weize Yuan, Remi Casier, Jean Duhamel

**Affiliations:** Institute for Polymer Research, Waterloo Institute of Nanotechnology, Department of Chemistry, University of Waterloo, Waterloo, ON N2L 3G1, Canada; w96yuan@mit.edu (W.Y.); remi.casier@uwaterloo.ca (R.C.)

**Keywords:** poly(glutamic acid), pyrene excimer fluorescence, fluorescence blob model, denaturation

## Abstract

The denaturation undergone by α–helical poly(*L*-glutamic acid) (P*L*GA) in *N*,*N*-dimethylformamide upon addition of guanidine hydrochloride (GdHCl) was characterized by comparing the fluorescence of a series of P*L*GA constructs randomly labeled with the dye pyrene (Py-P*L*GA) to that of a series of Py-P*DL*GA samples prepared from a racemic mixture of *D*,*L*-glutamic acid. The process of pyrene excimer formation (PEF) was taken advantage of to probe changes in the conformation of α–helical Py-P*L*GA. Fluorescence Blob Model (FBM) analysis of the fluorescence decays of the Py-P*L*GA and Py-P*DL*GA constructs yielded the average number (<*N*_blob_>) of glutamic acids located inside a *blob*, which represented the volume probed by an excited pyrenyl label. <*N*_blob_> remained constant for randomly coiled Py-P*DL*GA but decreased from ~20 to ~10 glutamic acids for the Py-P*L*GA samples as GdHCl was added to the solution. The decrease in <*N*_blob_> reflected the decrease in the local density of P*L*GA as the α–helix unraveled in solution. The changes in <*N*_blob_> with GdHCl concentration was used to determine the change in Gibbs energy required to denature the P*L*GA α–helix in DMF. The relationship between <*N*_blob_> and the local density of macromolecules can now be applied to characterize the conformation of macromolecules in solution.

## 1. Introduction

The Fluorescence Blob Model (FBM) was introduced in 1999 as a mathematical tool to extract quantitative information about the internal dynamics of polymers, that had been randomly labeled with the fluorescent dye pyrene [1]. As discussed in several reviews [2,3,4,5], the FBM takes advantage of the ability of an excited pyrene to form an excimer upon diffusive encounter with a ground-state pyrene inside the volume probed by the excited pyrene, and referred to as a *blob*. In turn, the *blob* could be used as a unit volume to divide the polymer coil into a cluster of identical subvolumes. Random labeling of a polymer with a pyrene derivative ensured that the pyrenyl labels would distribute themselves randomly among the *blobs* according to a Poisson distribution. Analysis of the fluorescence decays acquired with solutions of the pyrene-labeled polymers yielded the average number (<*n*>) of ground-state pyrenes per *blob*, which could be related to the number (*N*_blob_) of structural units encompassed inside a *blob*. For different polymers, a larger *N*_blob_ typically indicated that the excited pyrenyl label could probe a larger volume, reflecting a more flexible polymeric backbone.

While these early studies [1,2,3,4,5] established the FBM as an interesting means for gauging the flexibility of one polymer backbone against another, they overlooked an important aspect of the FBM, which is its ability to provide quantitative information about the local density of a macromolecule. In turn, this information could be related to the conformation of the macromolecule of interest, a most important research topic in macromolecular science. Based on this insight, a combination of pyrene excimer formation (PEF), FBM, and molecular mechanics optimizations (MMOs) was applied to yield the internal density of arborescent poly(*L*-glutamic acid)s (P*L*GAs) [6], confirm the helical conformation of amylose in dimethylsulfoxide (DMSO) [7], P*L*GA in *N*,*N*-dimethylformamide (DMF) [8,9,10], and poly(*L*-lysine) in acetonitrile/water mixtures [11], predict the 3_10_-helical conformation of P*L*GA in DMSO [12], introduce the Solution-Cluster model to describe the interior of amylopectin in DMSO [13,14], and provide the first 1:1 direct relationship between the experimental and predicted folding time of proteins [15,16].

The ability to characterize the conformation of macromolecules in solution makes *N*_blob_ a central parameter in the study of macromolecules by PEF [1,2,3,4,5], in the same manner as the radius of gyration (*R*_g_) and ellipticity (*θ*) are central parameters to, respectively, scattering [17,18,19] or circular dichroism (CD) [20,21,22,23,24,25,26] measurements to probe the conformation of, respectively, synthetic macromolecules or proteins in solution. To further assess the ability of *N*_blob_ to probe macromolecular conformations in solution, the present study investigates how *N*_blob_ reports on the unravelling of an α–helical P*L*GA in DMF as guanidine hydrochloride (GdHCl), a well-known denaturing agent [27], is added to the solution. To this end, a sample of P*L*GA and of poly(*D*,*L*-glutamic acid) (P*DL*GA) were randomly labeled with 1-pyrenemethylamine (PyMA) to yield Py-P*L*GA and Py-P*DL*GA, respectively, and PEF was applied to probe conformational changes in P*L*GA as GdHCl was added to the solution. P*L*GA is known to adopt an α–helical conformation in DMF [8,9,10], while the racemic nature of P*DL*GA imposes that it adopts a random coil conformation under any solvent condition. Consequently, P*DL*GA with its random coil conformation regardless of solvent conditions provided an ideal baseline against which the conformation of P*L*GA could be compared as GdHCl was added to the polypeptide solutions. The fluorescence decays of dilute solutions of Py-P*L*GA and Py-P*DL*GA in DMF were acquired and analyzed according to the FBM to retrieve *N*_blob_ as a function of the concentration of GdHCl added to the solutions. Comparison of the *N*_blob_ values obtained for the randomly coiled Py-P*DL*GA samples and the α–helical Py-P*L*GA samples in DMF without GdHCl provided a means to assess the extent of denaturation in the P*L*GA α–helix as GdHCl was added.

The results show that *N*_blob_ reflected the extent of the denaturation of the P*L*GA α–helix, decreasing as the helix unfolded with increasing GdHCl concentration, while *N*_blob_ remained constant for P*DL*GA in DMF over the entire GdHCl concentration range. The *N*_blob_ value was also employed to determine the molar fraction of native (*f*_N_) and denatured (*f*_D_) P*L*GA molecules in solution. In turn, the *f*_N_ and *f*_D_ fractions could be applied to determine the equilibrium constant (*K*^unfold^) for the Native ⇌ Denatured equilibrium at each GdHCl concentration [28,29] and yield the change in Gibbs energy (Δ_unfold_*G*(DMF)) for the unfolding of the α–helical P*L*GA in DMF in the same manner, that experiments using CD or LS would do. In summary, these PEF experiments further support the notion that *N*_blob_, determined for macromolecules randomly labeled with pyrene, reports on the local density of macromolecules and can be used to infer their conformation in solution.

## 2. Materials and Methods

### 2.1. Sample Preparation

**Figure 1:**
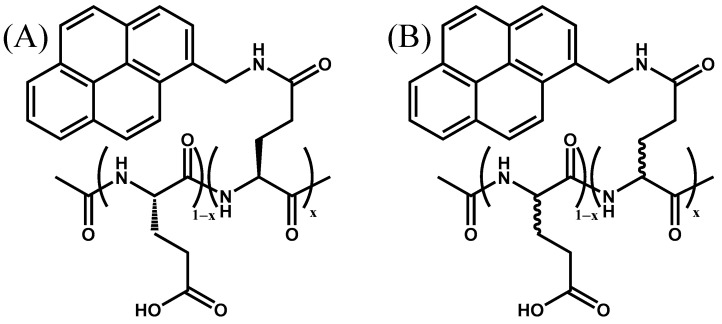
Chemical structure of (**A**) Py(*x*)-P*L*GA (*x* = 2.9, 4.4, 4.9, 6.9, 9.0, and 14.3 mol%) and (**B**) Py(*x*)-P*DL*GA (*x* = 6.0, 8.0, 10.4, 11.3, and 12.3 mol%) samples used in this study.

The preparation of the Py-P*L*GA and Py-P*DL*GA samples has been described earlier [10]. Six Py-P*L*GA samples and five Py-P*DL*GA samples were used in these experiments. Their chemical structure is described in Figure 1. The samples were dissolved in DMF (Sigma, ≥99.8%) before being diluted so that their concentration in pyrenyl label would equal 2.5 × 10^−6^ M, low enough to avoid any intermolecular interactions. Oxygen dissolved in the Py-P*L*GA solutions was outgassed by passing a gentle flow of 99.99% high purity N_2_ (Praxair, N4.0) for 30 min. Steady-state (SSF) and time-resolved (TRF) fluorescence experiments (SSF: LS-100 Photon Technology International, London, ON, Canada; TRF: IBH Ltd, Glasgow, Scotland, UK) were conducted with the degassed solutions.

### 2.2. Steady-State Fluorescence

A PTI spectrofluorometer was used to acquire the SSF spectra with a 344 nm excitation wavelength. The excitation and emission slit widths were set at 2 and 1 nm, respectively. The SSF spectrum was then analyzed by determining the fluorescence intensity of the monomer (*I*_M_) and excimer (*I*_E_) from the integration of the area under the spectrum from 372 to 378 nm and from 500 to 530 nm, respectively. These intensities were used to determine the *I*_E_/*I*_M_ ratio, which was employed to gauge the PEF efficiency.

### 2.3. Time-Resolved Fluorescence

The TRF decays were acquired with an IBH time-correlated single photon counting (TC-SPC) fluorometer using a 340 nm-NanoLED for excitation. The solutions were excited at 344 nm with an excitation monochromator and the monomer and excimer fluorescence decays were collected at 375 and 510 nm using a 370 and 495 nm cutoff filter, respectively. A repetition rate of 1 MHz or 500 kHz, time per channel of 1.02 or 2.04 ns/ch, and number of counts at the decay maximum of 40,000 or 20,000 counts were applied to the monomer and excimer fluorescence decays, respectively. These experimental settings were the same as those reported in earlier publications [10,12].

### 2.4. Fluorescence Decay Analysis

The FBM was employed to fit the TRF decays [1,2,3,4,5] using in-house software. The FBM assumes that five different pyrene species exist in solution. The species *Py*_diff_* represents the excited pyrenes, that diffuse toward a ground-state pyrene to yield the species *Py*_k2_*, where the excited and the ground-state pyrene labels are close enough to rearrange rapidly with a large rate constant *k*_2_ to form an excimer. The pyrenes, that result in excimer formation, are referred to as *E*0* or D* depending on whether the excimers produced are the result of the interaction between two well-stacked or two poorly stacked pyrenes, respectively. The fifth species, *Py*_free_* cannot form excimer and emits as if it were free in solution. The natural lifetime of the three species *Py*_diff_*, *Py*_k2_*, and *Py*_free_* is that of the pyrene monomer (*τ*_M_), whereas *E*0* and D* emit with their natural lifetimes *τ*_E0_ and *τ*_D_, respectively. The monomer and excimer fluorescence decays were fitted globally first with the program globmis90lbg, where *k*_2_ is allowed to float freely, and then with the program globmis90obg, where *k*_2_ is fixed in the analysis to its average value obtained with the earlier round of fits. The analysis provides the molar fractions *f*_diff_, *f*_k2_, *f*_free_, *f*_E0_, and *f*_D_ of the pyrene species *Py*_diff_*, *Py*_k2_*, *Py*_free_*, *E*0*, and D*, respectively. The sum of the fractions *f*_E0_ and *f*_D_ is referred to as *f*_agg_ since it represents the molar fraction of aggregated pyrenes. The FBM analysis also yields the average number <*n*> of ground-state pyrenes inside a *blob*, the product *k*_e_ × [*blob*] of the rate constant *k*_e_ for the exchange of ground-state pyrenes between *blobs* and the *blob* concentration [*blob*], and the rate constant *k*_blob_ for diffusive encounters between two pyrenyl labels inside a *blob*. All the parameters retrieved from the fit of the fluorescence decays were optimized with the Marquardt–Levenberg algorithm [30]. The equations used to fit the monomer and excimer fluorescence decays globally according to the FBM are provided as Supplementary Material (SM) as Equations (S1) and (S2), respectively, along with tables listing the FBM parameters retrieved from this analysis. In turn*,* the number of structural units found inside the volume of a *blob* (*N*_blob_) can be obtained from <*n*> according to Equation (1), where *x* is the molar fraction of glutamic acids, that were labeled with 1-pyrenmethylamine (see Figure 1), and *f*_Mfree_ represents the molar fraction of the *Py*_free_* species contributing to the monomer decay.

(1) $$N_{\mathrm{blob}}=\frac{<n>}{x}\left(1-f_{M\mathrm{free}e}\right)$$

## 3. Results

### 3.1. Steady-State Fluorescence

**Figure 2:**
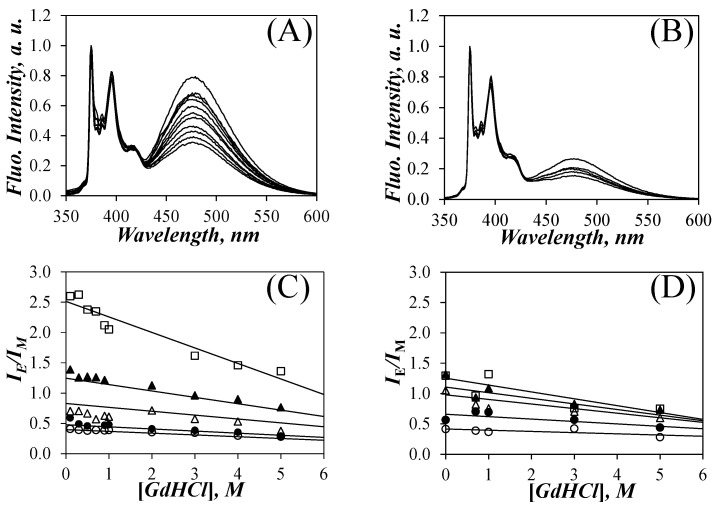
SSF spectra of (**A**) Py(14.0)-P*L*GA and (**B**) Py(10.4)-P*DL*GA in DMF with different GdHCl concentrations. From bottom to top: (**A**) [GdHCl] = 5.0, 4.0, 3.0, 2.0, 1.0, 0.9, 0.7, 0.5, 0.3, 0.1, 0.0 M and (**B**) [GdHCl] = 5.0, 3.0, 1.0, 0.7, and 0.0 M. Plots of *I*_E_/*I*_M_ for (**C**) Py(*x*)-P*L*GA, where *x* = (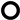) 4.4, (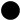) 4.9, (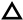) 6.9, (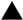) 9.0, and (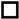) 14.3 mol%, and (**D**) Py(*x*)-P*DL*GA, where *x* = (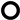) 6.0, (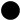) 8.0, (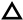) 10.4, (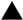) 11.3, and (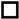) 12.3 mol%.

Since Py-P*L*GA adopts an α–helical conformation in DMF [8,9,10,31], the effect of GdHCl (Sigma, ≥99%), a well-known denaturing agent [27], on the denaturation of the P*L*GA helix was investigated by monitoring the fluorescence response of the Py-P*L*GA constructs as a function of the GdHCl concentration, which was varied from 0.1 to 5 M. The SSF spectra of solutions in DMF of five Py-P*L*GA and five Py-P*DL*GA samples were acquired for different GdHCl concentrations. The effect of the addition of GdHCl to the solution of Py(14.0)-P*L*GA and Py(10.4)-P*DL*GA in DMF is shown in Figure 2A,B after normalization at 375 nm, which corresponds to the 0–0 transition of pyrene. For both samples, the fluorescence of the pyrene excimer centered at 480 nm decreased with increasing GdHCl concentration. Although this effect was observed for all Py-PGA samples, it was more pronounced for the Py-P*L*GA samples. This is illustrated in a more quantitative manner by plotting the *I*_E_/*I*_M_ ratio as a function of [GdHCl] in Figure 2C,D.

The behavior of the *I*_E_/*I*_M_ ratio could be discussed in terms of Equation (2) [32], which shows how the *I*_E_/*I*_M_ ratio is related to the rate constant *k*_diff_ of diffusive encounters between an excited and a ground-state pyrenyl label and the local pyrene concentration [*Py*]_loc_. As described by Equation (2), the reduction in *I*_E_/*I*_M_ could be a result of one or more of the following three effects. First, the increase in viscosity by the addition of GdHCl is expected to reduce *k*_diff_. Second, the probability of PEF upon encounter between an excited and a ground-state pyrenyl label [33] might change with GdHCl concentration and is known to affect *k*_diff_. Third, the denaturation of the P*L*GA α–helix, which would reduce [*Py*]_loc_. The difficulty in identifying which one of these parameters best rationalized the effects observed with the *I*_E_/*I*_M_ ratios was resolved by applying the FBM analysis to the decays acquired with the pyrene-labeled samples. As a matter of fact, the FBM is designed to separate the contributions arising from *k*_diff_ and [*Py*]_loc_ with the parameters *k*_blob_ (=*k*_diff_ × (1/*V*_blob_), where *V*_blob_ is the *blob* volume and 1/*V*_blob_ represents the concentration equivalent to one ground-state pyrene inside a *blob*) and <*n*> (=[*Py*]_loc_ × *V*_blob_). <*n*> can then be used to determine the number *N*_blob_ of GA’s contained within each blob volume according to Equation (1) [1,2,3,4,5].

(2) $$\frac{I_{E}}{I_{M}}\propto k_{\mathrm{diff}}\times {\left[Py\right]}_{\mathrm{loc}}$$

### 3.2. Time-Resolved Fluorescence

The fluorescence decays of the pyrene monomer and excimer were acquired for all samples and fitted globally according to the FBM with Equations (S1) and (S2) in SI. The lifetime (*τ*_M_) of the pyrene monomer was estimated by fitting the fluorescence decays of Py(2.3)-P*L*GA with a sum of exponentials. The low pyrene content of this sample ensured that it would form little excimer so that its long-lived behavior reflected isolated pyrenyl labels, whose decay time was attributed to *τ*_M_. *τ*_M_ was found to decrease from 215 to 207 ns, when the GdHCl concentration was increased from zero to 0.1 M, before decreasing linearly with increasing GdHCl concentration from 207 to 190 ns from 0.1 and 5 M according to Equation (3). The lifetime *τ*_M_ was fixed in the fluorescence decay analysis to its value determined with Equation (3) for a given GdHCl concentration. This modest decrease in *τ*_M_ with increasing GdHCl concentration indicated that GdHCl is not an efficient quencher of pyrene.

*τ*_M_ (ns) = −3.33 × [GdHCl] + 207 (3)

**Figure 3:**
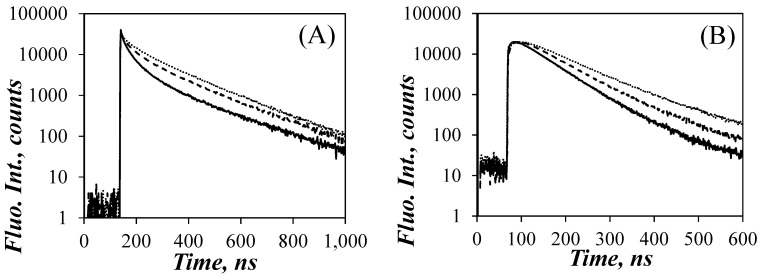
Fluorescence decays of the pyrene (**A**) monomer and (**B**) excimer of the Py(9.0)-P*L*GA solutions in DMF at different GdHCl concentrations. From bottom to top: [GdHCl] = 0.1, 1.0, 5.0 mol·L^−1^ yielding *N*_blob_ values of 19, 15, and 11, respectively.

The effect of GdHCl concentration on the monomer and excimer fluorescence decays can be seen in Figure 3, where the monomer and excimer fluorescence decays of Py(9.0)-P*L*GA are represented for GdHCl concentrations of 0.1, 1, and 5 M. The long-lived tails of the pyrene monomer decays in Figure 3A were essentially parallel, as expected since *τ*_M_ did not change much with GdHCl concentration. The early part of the monomer decay for Py(9.0)-P*L*GA in DMF with 0.1 M GdHCl showed a pronounced decrease reflecting efficient PEF, as would be expected if P*L*GA adopted a condensed conformation, such as that expected of an α–helix. This decrease in fluorescence intensity at the early times became less pronounced as more GdHCl was added to the solution, reflecting a decrease in PEF efficiency, that agreed with the *I*_E_/*I*_M_ trends observed in Figure 2C. The decrease in PEF observed for the monomer decays as more GdHCl was added to the solution was also observed in the excimer decays in Figure 3B, that showed a longer rise time with increasing GdHCl concentration. Similar to the SSF spectra in Figure 1A, the fluorescence decays also indicated that the addition of GdHCl affected PEF, suggesting that these changes might be related to the conformational changes experienced by the P*L*GA *a-*helix in DMF.

### 3.3. Fluorescence Blob Model Analysis of Decays

**Figure 4:**
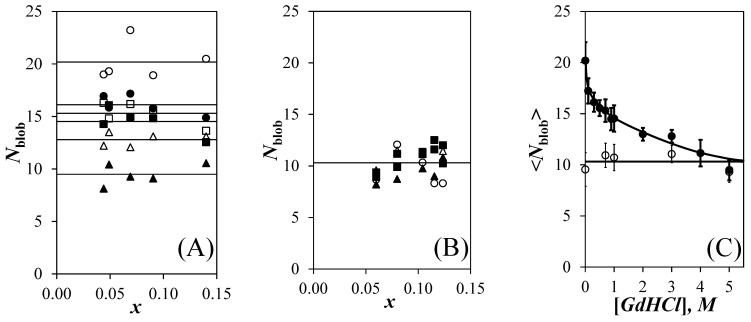
Plot of *N*_blob_ as a function of the mole fraction (*x*) of pyrene-labeled glutamic acids for (**A**) the Py-P*L*GA and (**B**) the Py-P*DL*GA samples in DMF with (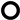) 0 M, (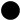) 0.3 M, (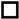) 0.7 M, (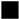) 1 M, (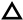) 3 M, and (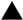) 5M GdHCl. (**C**) Plot of <*N*_blob_> as a function of GdHCl concentration for (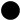) P*L*GA and (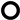) P*DL*GA.

After determining the *k*_2_ value at each GdHCl concentration, the *k*_2_ value was fixed for a given GdHCl concentration and the fluorescence decays were fitted according to Equations (S1) and (S2). The *N*_blob_ values were determined by introducing the <*n*> values retrieved from the fluorescence decay analysis into Equation (1). The *N*_blob_ values obtained for the Py-P*L*GA and Py-P*DL*GA samples are plotted as a function of pyrene content for different GdHCl concentrations in Figure 4A,B, respectively. For each GdHCl concentration, and despite the scatter, the *N*_blob_ values clustered around a constant value indicating that the pyrene-labeling did not affect the behavior of the polymers. The main difference in behavior between the Py-P*L*GA and Py-P*DL*GA samples was that *N*_blob_ decreased continuously with increasing GdHCl concentration in Figure 4A while *N*_blob_ remained constant and equal to 10.4 (±1.3) for the Py-P*DL*GA samples in Figure 4B. This effect was clearly illustrated in Figure 4C, where the *N*_blob_ values obtained as a function of pyrene content were averaged to yield <*N*_blob_>, which was plotted as a function of GdHCl concentration. <*N*_blob_> decreased from 20.2 (±1.8) for Py-P*L*GA in DMF without GdHCl to an average value of 10.2 (±1.5) for Py-P*L*GA with 4 and 5 M GdHCl. An <*N*_blob_> value of 20.2 (±1.8) has been reported numerous times, when Py-P*L*GA adopts an α–helical conformation [9,10,12], as it is known to do in DMF [31]. With 4 and 5 M GdHCl, <*N*_blob_> for Py-P*L*GA approached the <*N*_blob_> value for randomly coiled Py-P*DL*GA in DMF, that remained constant and equal to 10.4 (±1.3) at all GdHCl concentrations. Overall, the results in Figure 4C indicate that addition of GdHCl to a solution of α–helical P*L*GA in DMF induces the progressive unraveling of the P*L*GA α–helix, until it becomes a random coil at high GdHCl concentrations.

**Figure 5:**
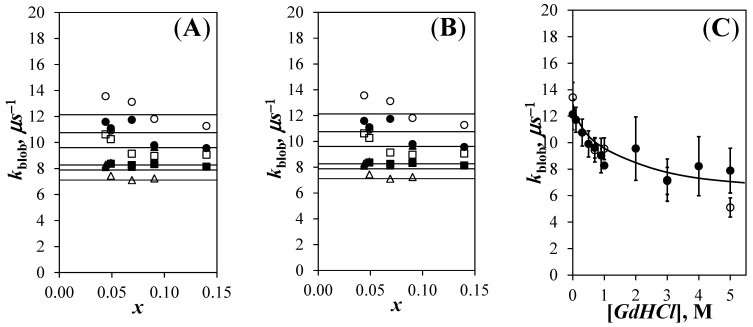
Plot of *k*_blob_ as a function of the mole fraction (*x*) of pyrene-labeled glutamic acids for (**A**) the Py-P*L*GA and (**B**) the Py-P*DL*GA samples in DMF with (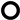) 0 M, (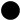) 0.3 M, (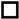) 0.7 M, (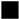) 1 M, (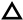) 3 M, and (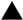) 5 M GdHCl. (**C**) Plot of *k*_blob_ as a function of GdHCl concentration for (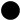) P*L*GA and (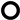) P*DL*GA.

The FBM analysis of the fluorescence decays also yielded *k*_blob_, which was plotted as a function a pyrene content in Figure 5A,B for the Py-P*L*GA and Py-P*DL*GA samples, respectively. In agreement with *N*_blob_, *k*_blob_ did not change much with pyrene content within experimental error, again implying that the pyrene content did not affect the behavior of the polymers. The *k*_blob_ values obtained as a function of pyrene content in Figure 5A,B were averaged to yield <*k*_blob_>, which was plotted as a function of GdHCl concentration in Figure 5C. Within experimental error, the <*k*_blob_> values for the Py-P*L*GA and Py-P*DL*GA samples showed a similar trend, with *<k*_blob_> decreasing with increasing GdHCl concentration. The decrease in *k*_blob_ was most certainly a consequence of the increase in the solution viscosity associated with the addition of fairly large amounts of GdHCl and the main contributor to the decrease in the *I*_E_/*I*_M_ ratios observed for the Py-P*DL*GAs in Figure 2D.

The trends shown in Figure 4C and Figure 5C displayed some remarkable features. A decrease in <*N*_blob_>, such as that displayed by the Py-P*L*GA samples in Figure 4C, is normally associated with smaller *V*_blob_ and larger <*k*_blob_> values, since *k*_blob_ = *k*_diff_ × (1/*V*_blob_). The decrease of both <*N*_blob_> in Figure 4C and <*k*_blob_> in Figure 5C for the Py-P*L*GA samples with increasing GdHCl concentration was thus noticeable. The constancy of *N*_blob_ with GdHCl concentration found for the Py-P*DL*GA samples suggested that *V*_blob_ did not change. Therefore, the ~30% decrease in *k*_blob_ observed for Py-P*DL*GA must have been a consequence of the increase in viscosity associated with the addition of large amounts of GdHCl. The similarity of the *k*_blob_-*vs*-[GdHCl] trends in Figure 5C for both Py-P*L*GA and Py-P*DL*GA, coupled with the fact that they were labeled with pyrene in the same manner, suggested that both samples shared a same *V*_blob_, which remained constant over the range of GdHCl concentrations studied. This implied that the decrease in *N*_blob_ found for Py-P*L*GA in Figure 4C with increasing GdHCl concentration reflected a decrease in the local polypeptide concentration experienced by an excited pyrenyl label, as expected with the unravelling of the P*L*GA α–helix. The reduction in the local peptide concentration as Py-P*L*GA transitions from an α–helix to a random coil also leads to a reduction in [Py]_loc_, which explains the more pronounced change in the *I*_E_/*I*_M_-vs-[GdHCl] trends observed for Py-P*L*GA than those observed for Py-PDLGA in Figure 2C,D, respectively.

In summary, *N*_blob_ appeared to be a direct measure of the number of structural units encompassed inside *V*_blob_. Since *V*_blob_ did not change with GdHCl concentration, *N*_blob_ thus reflected the local density of the polypeptides in solution. This conclusion is supported by earlier reports, which also suggested that FBM experiments report directly on the local density of a polypeptide as experienced by an excited pyrenyl label [15,16]. Consequently, the results obtained up to this point suggested that the *N*_blob_ values reported in Figure 4C reflected the extent of structured P*L*GA existing in the solution, and could possibly be handled in the same manner as other structural parameters commonly used to gauge the structural content of polypeptides in solution such as ellipticity [20,21,22,23,24,25], fluorescence intensity [25,26], or fluorescence anisotropy [26]. The implication of this conclusion was that *N*_blob_ could be employed to probe the stability of a polypeptide upon addition of a denaturant. These considerations are discussed hereafter.

## 4. Discussion

### 4.1. Unfolding of a Protein According to the Two-State Model

The stability of a protein is usually defined by its ability to resist unfolding upon being subject to denaturing forces resulting from the addition of a denaturing agent, a sudden change in solution pH, or a step increase in temperature [28,29,34]. A quantitative measure of the stability of a protein is provided by the change in Gibbs energy (Δ_unfold_*G*(H_2_O)) between the folded (*N*) and fully unfolded (*D*) conformation of a protein in water. Numerous reports in the literature suggest that the folding of many proteins follows the two-state model, whereby an equilibrium exists between the denatured and native state of proteins, as shown in Equation (4) [28,29].

(4) $$\begin{array}{c} K^{\mathrm{unfold}}\\ N\;D \end{array}$$

The equilibrium constant *K*^unfold^ describing the equilibrium between the native and unfolded protein can be related to Δ_unfold_*G*(H_2_O) according to Equation (5), where *f*_D_ and *f*_N_ represent the molar fractions of denatured and native protein, respectively. In turn, the ratio *f*_D_/*f*_N_ is expected to be well represented by an experimental observable (*Y*), that accurately reflects the extent of denaturation experienced by the protein of interest.

(5) $$K^{\mathrm{unfold}}=\exp\left(-\frac{\Delta_{\mathrm{unfold}}G\left(H_{2}O\right)}{RT}\right)=\frac{f_{D}}{f_{N}}=\frac{Y-Y_{N}}{Y_{D}-Y}$$

Based on the Linear Extrapolation Method (LEM) first introduced by Green and Pace [20], a plot of *Ln*(*f*_D_/*f*_N_) should decrease linearly with the denaturant concentration as shown in Equation (6), with the *y*-intercept yielding Δ_unfold_*G*(H_2_O). In water, Δ_unfold_*G*(H_2_O) values around ~40 kJ·mol^−1^ have been reported for ~300 *aa*-long proteins [29]. The slope (*m*) is related to the ability of the denaturant to unfold a protein and would take a value of ~4 kJ·mol^−1^·M^−1^ [29].

(6) $$-Ln(f_{D}/f_{N})=\Delta_{\mathrm{unfold}}G\left(DMF\right)-m\times \left[GdHCl\right]$$

### 4.2. Using <N_blob_> as a Structural Parameter

**Figure 6:**
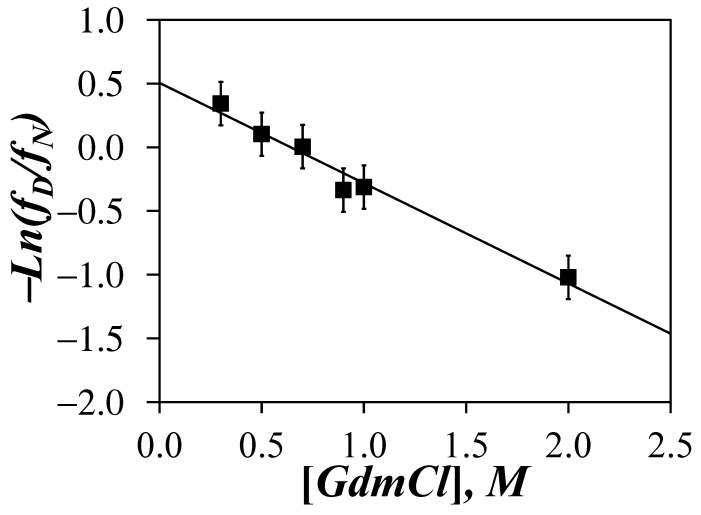
Plot of −*Ln*(*f*_D_/*f*_N_) as a function of GdHCl concentration where *f*_D_ and *f*_N_ are calculated from the <*N*_blob_> values obtained for Py-P*L*GA trend shown in Figure 4C.

Since the <*N*_blob_>-*vs*-[GdHCl] trends obtained in Figure 4C suggested that <*N*_blob_> reflected the structural content of the Py-P*L*GA samples in DMF as a function of GdHCl concentration, we decided to investigate whether <*N*_blob_> could be taken as such an observable (i.e., *Y* = <*N*_blob_> in Equation (5)) to determine Δ_unfold_*G*(DMF) for the unfolding of an α–helical P*L*GA in DMF into a random coil upon addition of GdHCl. We also note that while the ellipticity of a protein determined by circular dichroism is normally the observable of choice to determine Δ_unfold_*G*(H_2_O), the strong amide absorption of DMF would rule out the use of CD to determine Δ_unfold_*G*(DMF) for the unfolding of a protein in DMF. The <*N*_blob_> values obtained in Figure 4C were introduced into Equation (5) to determine the *f*_D_/*f*_N_ ratio for GdHCl concentrations between 0.3 and 2 M, using an <*N*_blob_> value of 20.2 and 10.4 obtained for α–helical Py-P*L*GA in DMF without GdHCl and randomly coiled Py-P*DL*GA in DMF over all GdHCl concentrations for the *Y*_N_ and *Y*_D_ values, respectively. −*Ln*(*f*_D_/*f*_N_) was plotted as a function of GdHCl concentration in Figure 6. A satisfactory straight line was obtained with an intercept corresponding to a Δ_unfold_*G*(DMF) value of 1.3 (±0.2) kJ·mol^−1^ and an *m* value of 1.9 (±0.2) kJ·mol^−1^·M^−1^.

Application of the LEM using <*N*_blob_> as an experimental observable to measure Δ_unfold_*G*(DMF) for the unfolding of P*L*GA in DMF upon the addition of GdHCl resulted in a surprisingly good linearity between −*Ln*(*f*_D_/*f*_N_) and the GdHCl concentration in Figure 6. The Δ_unfold_*G*(DMF) value of 1.3 (±0.2) kJ·mol^−1^ obtained from the *y*-intercept of the plot was more than one order of magnitude lower than the Δ_unfold_*G*(H_2_O) values reported for the unfolding of globular proteins in water [29]. Such a difference between Δ_unfold_*G*(DMF) and Δ_unfold_*G*(H_2_O) was to be expected. Beside the fact that DMF was used instead of H_2_O, the main difference in stability between P*L*GA and proteins was most likely due to the extended conformation of α–helical P*L*GA, which could not benefit from the many additional stabilizing interactions existing between the structural motives found in the interior of globular proteins in water [35,36,37,38]. Instead, internal H-bonds between the amide bonds of the polypeptide backbone were the only stabilizing contributions to the structural integrity of the P*L*GA α–helix [39], which were easily neutralized by the addition of GdHCl. These considerations rationalize the rather low Δ_unfold_*G*(DMF) value obtain for P*L*GA in DMF. Although low, the *m* value of 1.9 (±0.2) kJ·mol^−1^·M^−1^ retrieved for P*L*GA was only half the value expected for globular proteins, suggesting that the unfolding of the P*L*GA α–helix in DMF results in substantial exposure of the glutamic acid residues to the solvent.

### 4.3. Strengths and Weaknesses of PEF-Based Macromolecular Structure Determination

As already discussed in earlier works [7,8,9,10,11,12], the FBM analysis of the PEF signal generated by macromolecules randomly labeled with pyrene yields the parameter *N*_blob_, which describes the conformation of structured macromolecules in solution. The fact that the macromolecule needs to be randomly labeled does not require specific attachment points, and can be polydisperse represents important advantages to the method. It also takes advantage of the outstanding sensitivity of fluorescence to probe macromolecules under extremely dilute conditions, typically at concentrations around 1 mg/L, two-to-three orders of magnitude lower than most other standard techniques used for structure determination like scattering or NMR. Despite its formidable advantages, the PEF-based method also has some important disadvantages, which should not be overlooked. First, pyrene is hydrophobic and aggregates in water, making the structural study of pyrene-labeled macromolecules in water challenging [40]. Second, the random labeling of a macromolecule is well suited to characterize its structure in solution as long as the pyrenyl labels are attached at its periphery such as onto the side groups of helices of amylose [7] or P*L*GA or poly(*L*-lysine) [8,9,10,11,12]. In the case of a protein containing several closely packed structural motives, the random introduction of pyrenyl labels onto the motives would interfere with their tight packing, which would affect the structure of the protein. Third, a PEF experiment reports on a macromolecular structure over a length scale, that is defined by the reach of a pyrenyl label bound to the macromolecule via a linker of specific length. In the case of the PGAs randomly labeled with 1-pyrenemethylamine, the maximum distance separating two α–carbon in the polypeptide backbone would equal 3.1 (±0.2) and 3.1 (±0.4) nm for α–helical P*L*GA and randomly coiled P*DL*GA constructs, which corresponded to <*N*_blob_> values of 20.5 (±1.5) and 10.5 (±1.5) in DMF, respectively. Fourth, polymers containing chemical groups such as amines [11] or primary amides (but not secondary or tertiary amides) capable of quenching the pyrene fluorescence cannot be studied. Nevertheless, and despite these drawbacks, many synthetic and natural polymers remain, whose characterization would strongly benefit from the determination of their conformation in solution through a PEF study.

## 5. Conclusions

A series of experiments were conducted, where the PEF of Py-P*L*GA and Py-P*DL*GA constructs in DMF was analyzed with the FBM to yield <*N*_blob_> as a function of the amount of GdHCl, a known denaturing agent [27], that was added to the solution. <*N*_blob_> decreased progressively from a value of 20.2 (±1.8) for Py-P*L*GA in DMF without GdHCl to 10.2 (±1.5) in DMF with 4 or 5 M GdHCl. Since <*N*_blob_> values of ~20 and ~10 are those expected for α–helical and randomly coiled Py-P*L*GA, respectively [9,10,12], the <*N*_blob_>-*vs*-[GdHCl] trend obtained for Py-P*L*GA in Figure 4C was taken as evidence that these FBM experiments reflected the unravelling of the P*L*GA α–helix as GdHCl was added to the solution. Furthermore, the constancy of <*N*_blob_> observed for the Py-P*DL*GA samples suggested that the *blob* volume (*V*_blob_) remained constant with GdHCl concentration and that the decrease in *k*_blob_ with increasing GdHCl concentration observed for Py-P*DL*GA must have been due to an increase in solution viscosity with increasing GdHCl concentration. Combining the constancy in *V*_blob_ with the similarity of the *k*_blob_-*vs*-[GdHCl] plots obtained for the Py-P*L*GA and Py-P*DL*GA samples led to the conclusion, that *V*_blobs_ remained constant for the Py-P*L*GA samples and that *N*_blob_ reflected the change in polymer density experienced by an excited pyrenyl label as the P*L*GA α–helix unraveled upon addition of GdHCl. This conclusion agreed with those reached in earlier studies [15,16] and represents an important improvement in the applicability of the FBM to probe the local density of macromolecules in solution, a feature that used to be mainly accessible by scattering techniques.

The inferred ability of <*N*_blob_> to report on the extent of structural content of the P*L*GA α–helix was further confirmed by applying the LEM to determine the change in Gibbs energy (Δ_unfold_*G*(DMF)) for the unfolding of P*L*GA in DMF upon addition of GdHCl. The good linearity observed in Figure 6 between −*Ln*(*f*_D_/*f*_N_) and the GdHCl concentration suggested that <*N*_blob_> reported accurately on the structural content of P*L*GA. The low Δ_unfold_*G*(DMF) value retrieved from this analysis was mostly a consequence of dealing with an isolated α–helix, whose stability was the result of intramolecular H-bonding between the backbone amides [39]. These would represent fairly weak interactions compared to those experienced by the different structural motives inside a globular proteins [35,36,37,38], which must contribute to the higher Δ_unfold_*G*(H_2_O) values obtained for the unfolding of proteins in aqueous solutions.

In summary, this study provides further support to the notion that a combination of PEF and FBM analysis of pyrene-labeled macromolecules yields information about the density of macromolecules in solution. Because PEF occurs locally over ~3 nm in the case of Py-PGA constructs, the ability to use *N*_blob_ to probe the density of macromolecules over a ~3 nm length scale offers a means to probe macromolecules in solution at close range, a feature that should nicely complement the studies of macromolecules by scattering techniques, that typically probe entire macromolecules. Consequently, the PEF study of pyrene-labeled macromolecules opens new venues of research to characterize the conformation of complex macromolecules in solution.

## Data Availability

The data presented in this study are available on request from the corresponding author.

## Supplementary Materials

The following are available online at https://www.mdpi.com/article/10.3390/polym13111690/s1, FBM equations, plot of *k*_2_ as a function of GdHCl concentration, tables of parameters retrieved from the FBM of the fluorescence decays acquired with the Py-P*L*GA and Py-P*DL*GA samples.
