# Supplementary material for: Unfolding of Helical Poly(L-Glutamic Acid) in N,N-Dimethylformamide Probed by Pyrene Excimer Fluorescence (PEF)
Source: Polymers (Basel). 2021 May 22;13(11):1690. doi: 10.3390/polym13111690 (PMC8196828; doi:10.3390/polym13111690)
Supplement: Supplementary file 1 [file polymers-13-01690-s001.zip › polymers-1208101-supplementary.pdf]

polymers

MDPI

Article

Supplementary Material

Unfolding of Helical Poly(*L*-Glutamic Acid) in *N*,*N*-Dimethylformamide Probed by Pyrene Excimer Fluorescence (PEF)

Weize Yuan, Remi Casier and Jean Duhamel *

Institute for Polymer Research, Waterloo Institute of Nanotechnology, Department of Chemistry, University of Waterloo, Waterloo, ON N2L 3G1, Canada: w96yuan@mit.edu (W.Y.); remi.casier@uwaterloo.ca (R.C.)
* Correspondence: jduhamel@uwaterloo.ca; Tel.: +1-519-888-4567 (ext. 35916)

Table of Contents

(A) Equations Used to Analyze the Fluorescence Decays of Py-P*L*GA and Py-P*DL*GA. 1

(B) Example Global-Fit of the Monomer and Excimer Decays According to the FBM 3

(C) Parameters Retrieved Using the FBM Analysis 4

(A) Equations Used to Analyze the Fluorescence Decays of Py-P*L*GA and Py-P*DL*GA.

The monomer and excimer decays of the pyrene-labeled PGAs were fit with Equations (S1) and (S2), respectively.

$$[Py^*]_{(t)} = [Py^*_{diff}]_{(t)} + [Py^*_{k2}]_{(t)} + [Py^*_{free}]_{(t)} = [Py^*_{diff}]_o \exp\left(-\left(A_2+\frac{1}{\tau_M}\right)t - A_3\left(1-\exp(-A_4 t)\right)\right)$$

$$+\left([Py^*_{k2}]_o + [Py^*_{diff}]_o e^{-A_3}\sum_{i=0}^{\infty}\frac{A_3^i}{i!}\frac{A_2+iA_4}{A_2+iA_4-k_2}\right)\exp\left(-\left(k_2+\frac{1}{\tau_M}\right)t\right)$$

$$-[Py^*_{diff}]_o e^{-A_3}\sum_{i=0}^{\infty}\frac{A_3^i}{i!}\frac{A_2+iA_4}{A_2+iA_4-k_2}\exp\left(-\left(A_2+iA_4+\frac{1}{\tau_M}\right)t\right)$$

$$+[Py^*_{free}]_o \exp\left(-\frac{t}{\tau_M}\right)\Bigg] \qquad \text{(S1)}$$

$$[E^*]_{(t)} = [E0^*]_{(t)} + [D^*]_{(t)} + [ES^*]_{(t)} = k_2\left(\left([Py^*_{k2}]_o + [Py^*_{diff}]_o e^{-A_3}\sum_{i=0}^{\infty}\frac{A_3^i}{i!}\frac{A_2+iA_4}{A_2+iA_4-k_2}\right)\right.$$

$$\times \frac{\exp\left(-\frac{t}{\tau_{E0}}\right) - \exp\left(-\left(k_2 + \frac{1}{\tau_M}\right)t\right)}{k_2 + \frac{1}{\tau_M} - \frac{1}{\tau_{E0}}}$$

$$\left. + [Py_{diff}^{*}]_o e^{-A_3} \sum_{i=0}^{\infty} \frac{A_3^i}{i!} \frac{A_2 + iA_4}{A_2 + iA_4 - k_2} \frac{\exp\left(-\left(A_2 + iA_4 + \frac{1}{\tau_M}\right)t\right) - \exp\left(-\frac{t}{\tau_{E0}}\right)}{A_2 + iA_4 + \frac{1}{\tau_M} - \frac{1}{\tau_{E0}}} \right)$$

$$+ k_2 \left(\left([Py_{k2}^{*}]_o + [Py_{diff}^{*}]_o e^{-A_3} \sum_{i=0}^{\infty} \frac{A_3^i}{i!} \frac{A_2 + iA_4}{A_2 + iA_4 - k_2} \right) \times \frac{\exp\left(-\frac{t}{\tau_D}\right) - \exp\left(-\left(k_2 + \frac{1}{\tau_M}\right)t\right)}{k_2 + \frac{1}{\tau_M} - \frac{1}{\tau_D}} \right.$$

$$\left. + [Py_{diff}^{*}]_o e^{-A_3} \sum_{i=0}^{\infty} \frac{A_3^i}{i!} \frac{A_2 + iA_4}{A_2 + iA_4 - k_2} \frac{\exp\left(-\left(A_2 + iA_4 + \frac{1}{\tau_M}\right)t\right) - \exp\left(-\frac{t}{\tau_D}\right)}{A_2 + iA_4 + \frac{1}{\tau_M} - \frac{1}{\tau_D}} \right)$$

$$+ [E0^*]_o \times \exp\left(-\frac{t}{\tau_{E0}}\right) + [D^*]_o \times \exp\left(-\frac{t}{\tau_D}\right) \quad \text{(S2)}$$

In Equations (S1) and (S2), the parameters A_2, A_3, and A_4 are given in Equation S3.a-c.

$$A_2 = \langle n \rangle \times \frac{k_{blob} k_e [blob]}{k_{blob} + k_e [blob]} \quad \text{(S3.a)}$$

$$A_3 = \langle n \rangle \times \left(\frac{k_{blob}}{k_{blob} + k_e [blob]} \right)^2 \quad \text{(S3.b)}$$

$$A_4 = k_{blob} + k_e [blob] \quad \text{(S3.c)}$$

A full description of the parameters in Equations (S1)–(S3) is provided in the Experimental section of the main text. Equation S1 yields the molar fractions f_{Mdiff}, f_{Mk2}, and f_{Mfree} of the pyrene species Py_{diff}*, Py_{k2}*, and Py_{free}*, where the M-subscript indicates that these pyrene species were detected in the monomer fluorescence decays. Analysis of the excimer fluorescence decays with Equation S2 yields the molar fractions $f_{EdiffE0}$, f_{EdiffD}, f_{Ek2E0}, f_{Ek2D}, f_{EE0}, and f_{ED} of the pyrene species Py_{diffE0}*, Py_{diffD}*, Py_{k2E0}*, Py_{k2D}*, $E0$*, and D*, where the E-subscript indicates that these pyrene species were detected in the excimer fluorescence decays. The molar fractions obtained from the monomer or the excimer fluorescence decays were used to determine the molar fractions f_{diff}, f_{k2}, f_{free}, f_{E0}, and f_D whose expressions are given in Equations (S4)–(S8).

$$f_{diff} = \frac{1}{1 + \dfrac{f_{Mk2} + f_{Mfree}}{f_{Mdiff}} + \dfrac{f_{EE0} + f_{ED}}{f_{EdiffE0} + f_{EdiffD}}} \tag{S4}$$

$$f_{k2} = f_{diff} \times \frac{f_{Mk2}}{f_{Mdiff}} = f_{diff} \times \frac{f_{Ek2E0} + f_{Ek2D}}{f_{EdiffE0} + f_{EdiffD}} \tag{S5}$$

$$f_{free} = f_{diff} \times \frac{f_{Mfree}}{f_{Mdiff}} \tag{S6}$$

$$f_{E0} = f_{diff} \times \frac{f_{EE0}}{f_{EdiffE0} + f_{EdiffD}} \tag{S7}$$

$$f_{D} = f_{diff} \times \frac{f_{ED}}{f_{EdiffE0} + f_{EdiffD}} \tag{S8}$$

(B) Example Global-Fit of the Monomer and Excimer Decays According to the FBM

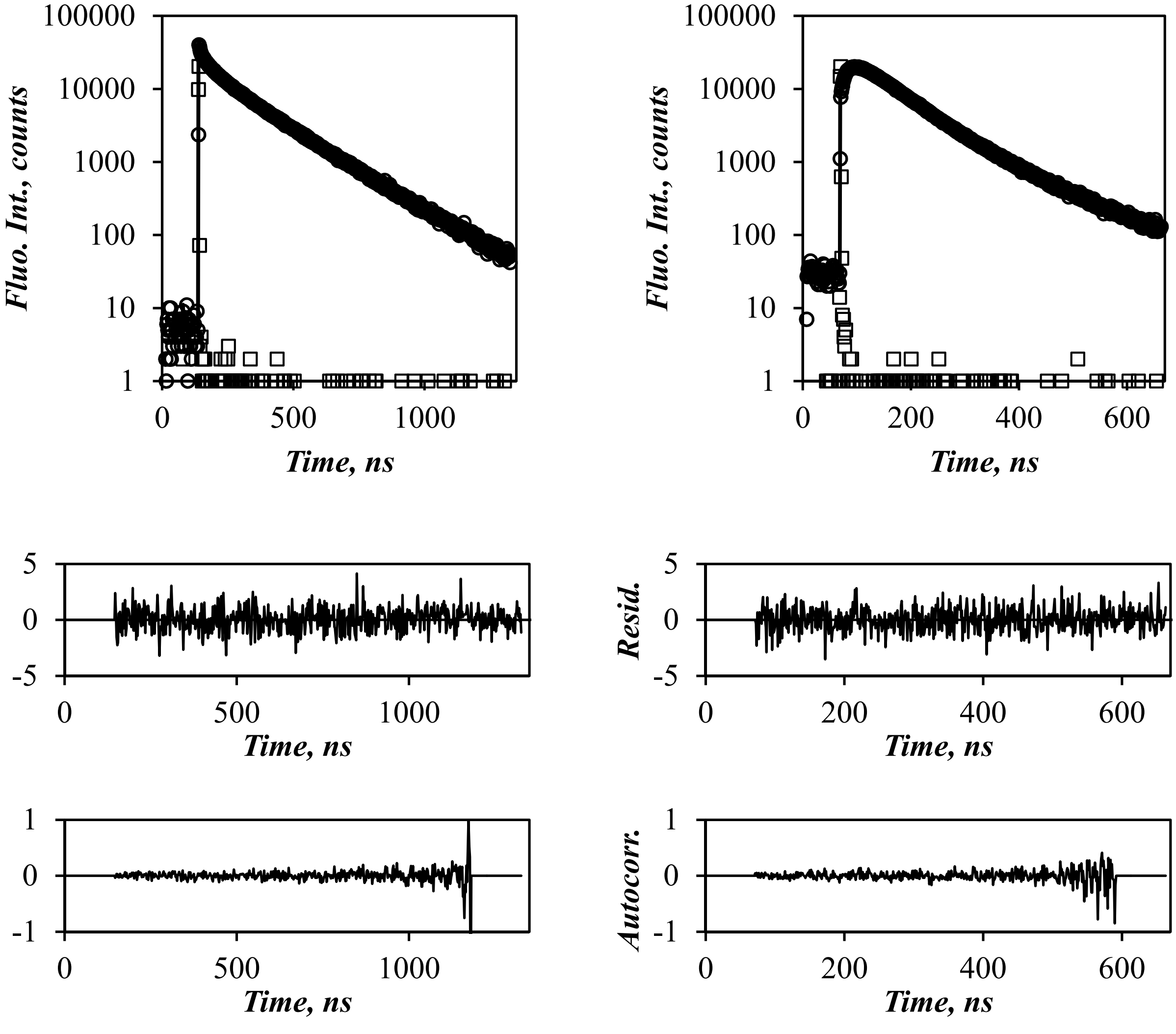

Figure S1. Monomer (left, λ_{em} = 375 nm) and excimer (right, λ_{em} = 510 nm) fluorescence decays (□) of Py(7.7)-P*L*GA in DMF containing 1.0 M GdHCl. The instrument response function (○) and the fit of the global analysis of the FBM (—) are overlaid with the fluorescence decays. λ_{ex} = 344 nm, [Py] = 2.5 × 10^{-6} M. χ^2 = 1.10.

(C) Parameters Retrieved Using the FBM Analysis

Table S1. Parameters retrieved using the FBM analysis of the monomer decays of Py(*x*)-P*L*GA in DMF solutions containing GdHCl.

[GdHCl] (M)	τ_M (ns)	k_2 (ns^{-1})	*x* (mol%)	f_{Mk2}	$k_e[blob]$ (μs^{-1})	<*n*>	k_{blob} (μs^{-1})	f_{Mdiff}	f_{Mfree}	χ^2
0.0	215	0.147	0.1	0.47	4.2	3.12	11.3	0.44	0.08	1.06
			4.4	0.21	3.5	1.12	13.6	0.51	0.25	1.14
			4.9	0.21	5.0	1.15	10.9	0.61	0.18	1.12
			6.9	0.35	5.3	1.80	13.1	0.52	0.11	1.04
			9.0	0.35	3.3	1.83	11.8	0.59	0.06	1.11
0.1	207	0.119	0.1	0.48	3.4	2.53	11.1	0.41	0.12	1.05
			4.4	0.21	1.0	1.14	11.7	0.44	0.36	1.18
			4.9	0.20	4.9	1.15	11.1	0.62	0.18	1.12
			6.9	0.36	4.1	1.47	11.5	0.44	0.20	1.21
			9.0	0.39	3.5	1.75	13.3	0.50	0.11	1.03
0.3	206	0.102	0.1	0.55	1.9	2.24	9.6	0.38	0.07	1.11
			4.4	0.21	2.3	1.26	11.6	0.38	0.41	1.09
			4.9	0.20	2.8	1.22	11.1	0.44	0.36	1.14
			6.9	0.33	6.8	1.44	11.7	0.50	0.18	1.16
			9.0	0.40	2.4	1.60	9.8	0.49	0.11	1.01
0.5	206	0.092	0.1	0.55	2.0	2.17	8.8	0.39	0.07	1.05
			4.4	0.23	1.3	1.18	11.3	0.39	0.38	1.14
			4.9	0.21	2.8	1.16	10.5	0.44	0.35	1.11
			6.9	0.27	3.5	1.34	9.6	0.53	0.19	1.11
			9.0	0.35	3.1	1.62	9.3	0.51	0.14	1.03
0.7	205	0.085	0.1	0.52	2.5	2.11	9.1	0.38	0.09	1.08
			4.4	0.21	2.0	1.23	10.6	0.37	0.42	1.15
			4.9	0.21	2.7	1.11	10.3	0.44	0.35	1.11
			6.9	0.28	3.1	1.47	9.1	0.48	0.24	1.11
			9.0	0.37	3.0	1.63	9.0	0.49	0.13	1.21
0.9	204	0.079	0.1	0.43	2.3	2.08	8.4	0.46	0.11	1.04
			4.4	0.22	1.2	0.95	11.3	0.41	0.36	1.09
			4.9	0.22	2.2	1.20	8.5	0.42	0.36	1.06
			6.9	0.29	3.2	1.34	8.9	0.45	0.27	1.02
			9.0	0.38	2.3	1.62	8.0	0.49	0.13	1.19
1.0	204	0.077	0.1	0.43	1.9	1.97	8.1	0.46	0.11	1.08
			4.4	0.23	1.3	1.04	11.3	0.37	0.40	1.06
			4.9	0.21	2.3	1.26	8.4	0.41	0.37	1.10
			6.9	0.31	2.7	1.34	8.3	0.46	0.23	1.23
			9.0	0.39	2.7	1.53	8.3	0.48	0.13	1.23
[GdHCl] (M)	τ_M (ns)	k_2 (ns^{-1})	*x* (mol%)	f_{Mk2}	$k_e[blob]$ (μs^{-1})	<*n*>	k_{blob} (μs^{-1})	f_{Mdiff}	f_{Mfree}	χ^2
2.0	200	0.061	0.1	0.51	4.5	2.02	6.5	0.38	0.11	1.11
			4.4	0.21	1.3	0.86	10.0	0.42	0.36	1.08
			4.9	0.24	3.4	1.03	11.0	0.36	0.40	1.13

			6.9	0.34	4.7	1.13	12.4	0.47	0.20	1.19
			9.0	0.37	2.8	1.47	7.8	0.49	0.14	1.02
3.0	197	0.052	0.1	0.52	4.5	2.04	5.5	0.38	0.10	1.17
			4.4	0.22	0.9	0.79	8.3	0.45	0.32	1.11
			4.9	0.22	3.6	1.10	7.4	0.38	0.40	1.09
			6.9	0.30	2.8	1.09	7.1	0.46	0.23	1.10
			9.0	0.39	2.9	1.37	7.2	0.47	0.14	1.04
4.0	194	0.045	0.1	0.54	2.9	1.62	6.2	0.40	0.06	1.19
			4.4	0.22	0.9	0.56	9.1	0.53	0.25	1.07
			4.9	0.21	6.9	1.13	7.4	0.33	0.46	1.11
			6.9	0.30	6.2	0.93	11.7	0.46	0.24	1.14
			9.0	0.35	3.0	1.33	6.7	0.51	0.14	1.08
5.0	191	0.040	0.1	0.46	0.9	1.55	5.1	0.49	0.05	1.06
			4.4	0.21	0.4	0.44	8.1	0.61	0.18	1.06
			4.9	0.22	6.2	0.93	8.4	0.33	0.45	1.27
			6.9	0.23	4.4	0.95	8.1	0.44	0.33	1.09
			9.0	0.40	8.5	1.06	9.7	0.38	0.22	1.14

Table S2. Parameters retrieved using the FBM analysis of the excimer decays of Py(x)-P*L*GA in DMF solutions containing GdHCl.

**[GdHCl] (M)**	k_2 **(ns⁻¹)**	x **(mol%)**	f_{Ek2}	τ_{E0} **(ns)**	$f_{EdiffE0}$	f_{EE0}	τ_D **(ns)**	f_{EdiffD}	f_{ED}	χ^2
0.0	0.147	0.1	0.35	41	0.18	0.33	69	0.14	0.00	1.06
		4.4	0.25	44	0.45	0.14	81	0.17	0.00	1.14
		4.9	0.22	44	0.48	0.11	82	0.18	0.00	1.12
		6.9	0.29	48	0.32	0.28	81	0.12	0.00	1.04
		9.0	0.33	41	0.35	0.07	68	0.22	0.04	1.11
0.1	0.119	0.1	0.34	48	0.23	0.37	79	0.06	0.00	1.05
		4.4	0.29	42	0.50	0.10	102	0.11	0.00	1.18
		4.9	0.22	42	0.46	0.11	78	0.22	0.00	1.12
		6.9	0.33	46	0.30	0.26	83	0.10	0.01	1.21
		9.0	0.29	49	0.29	0.33	83	0.09	0.00	1.03
0.3	0.102	0.1	0.47	45	0.25	0.17	69	0.08	0.03	1.11
		4.4	0.30	47	0.46	0.15	105	0.09	0.00	1.09
		4.9	0.28	42	0.47	0.10	89	0.15	0.00	1.14
		6.9	0.28	45	0.33	0.25	82	0.11	0.03	1.16
		9.0	0.37	46	0.31	0.17	76	0.14	0.00	1.01
0.5	0.092	0.1	0.45	47	0.25	0.23	71	0.07	0.00	1.05
		4.4	0.32	46	0.43	0.13	105	0.11	0.00	1.14
		4.9	0.29	44	0.46	0.11	90	0.14	0.00	1.11
		6.9	0.28	48	0.42	0.17	86	0.12	0.00	1.11
		9.0	0.36	41	0.44	0.01	75	0.08	0.12	1.03
0.7	0.085	0.1	0.42	47	0.23	0.27	71	0.08	0.01	1.08
		4.4	0.31	46	0.47	0.12	108	0.07	0.03	1.15
		4.9	0.30	43	0.45	0.10	86	0.15	0.00	1.11
		6.9	0.29	46	0.37	0.18	87	0.12	0.04	1.11
		9.0	0.37	44	0.36	0.14	72	0.13	0.00	1.21
0.9	0.079	0.1	0.31	47	0.25	0.35	72	0.08	0.00	1.04
		4.4	0.30	46	0.45	0.15	104	0.08	0.00	1.09

		4.9	0.31	45	0.49	0.10	97	0.11	0.00	1.06
		6.9	0.33	47	0.40	0.15	85	0.11	0.01	1.02
		9.0	0.38	46	0.38	0.13	79	0.11	0.00	1.19
		0.1	0.33	41	0.21	0.20	61	0.14	0.12	1.08
		4.4	0.33	46	0.41	0.15	97	0.08	0.00	1.06
1.0	0.077	4.9	0.30	45	0.49	0.10	99	0.10	0.00	1.10
		6.9	0.34	47	0.40	0.14	84	0.11	0.00	1.23
		9.0	0.39	46	0.35	0.13	72	0.13	0.00	1.23
		0.1	0.42	47	0.23	0.22	60	0.09	0.04	1.11
		4.4	0.29	45	0.49	0.11	106	0.07	0.04	1.08
2.0	0.061	4.9	0.33	49	0.38	0.17	97	0.10	0.02	1.13
		6.9	0.33	48	0.35	0.20	80	0.11	0.01	1.19
		9.0	0.34	43	0.32	0.19	69	0.14	0.00	1.02
		0.1	0.44	46	0.22	0.17	61	0.09	0.08	1.17
		4.4	0.28	49	0.51	0.14	124	0.06	0.01	1.11
3.0	0.052	4.9	0.32	50	0.46	0.13	104	0.08	0.00	1.09
		6.9	0.33	49	0.35	0.10	83	0.15	0.06	1.10
		9.0	0.36	45	0.31	0.19	73	0.13	0.01	1.04
		0.1	0.40	49	0.21	0.20	68	0.08	0.10	1.19
		4.4	0.25	49	0.53	0.12	118	0.07	0.03	1.07
4.0	0.045	4.9	0.30	57	0.44	0.24	159	0.03	0.00	1.11
		6.9	0.26	51	0.32	0.32	90	0.09	0.01	1.14
		9.0	0.31	47	0.32	0.21	77	0.13	0.02	1.08
		0.1	0.35	46	0.29	0.21	75	0.09	0.06	1.06
		4.4	0.22	52	0.57	0.13	136	0.07	0.01	1.06
5.0	0.040	4.9	0.32	57	0.42	0.16	121	0.07	0.02	1.27
		6.9	0.27	51	0.38	0.16	94	0.12	0.08	1.09
		9.0	0.34	59	0.29	0.33	119	0.03	0.00	1.14

Table S3. Global fractions of pyrene species calculated using parameters retrieved from the FBM analysis of Py(x)-P*L*GA in DMF solutions containing GdHCl.

**[GdHCl] (M)**	x **(mol%)**	f_{k2}	f_{diffE0}	f_{diffD}	f_{diff}	f_{E0}	f_D	f_{agg}	f_{free}	χ^2
	0.1	0.33	0.17	0.13	0.31	0.31	0.00	0.31	0.06	1.06
	4.4	0.19	0.34	0.13	0.47	0.10	0.00	0.10	0.23	1.14
0.0	4.9	0.19	0.40	0.15	0.56	0.09	0.00	0.09	0.16	1.12
	6.9	0.27	0.29	0.11	0.40	0.25	0.00	0.25	0.08	1.04
	9.0	0.31	0.33	0.21	0.53	0.06	0.04	0.10	0.06	1.11
	0.1	0.31	0.21	0.05	0.27	0.34	0.00	0.34	0.08	1.05
	4.4	0.19	0.34	0.07	0.41	0.07	0.00	0.07	0.33	1.18
0.1	4.9	0.18	0.38	0.18	0.56	0.09	0.00	0.09	0.16	1.12
	6.9	0.28	0.25	0.09	0.34	0.22	0.01	0.23	0.15	1.21
	9.0	0.27	0.27	0.08	0.35	0.30	0.00	0.30	0.08	1.03
	0.1	0.45	0.23	0.08	0.31	0.16	0.03	0.19	0.06	1.11
	4.4	0.19	0.29	0.06	0.35	0.09	0.00	0.09	0.37	1.09
0.3	4.9	0.19	0.31	0.10	0.41	0.06	0.00	0.06	0.34	1.14
	6.9	0.25	0.28	0.10	0.38	0.21	0.03	0.24	0.13	1.16
	9.0	0.34	0.28	0.13	0.41	0.16	0.00	0.16	0.09	1.01
0.5	0.1	0.43	0.24	0.07	0.30	0.22	0.00	0.22	0.05	1.05

	4.4	0.21	0.28	0.07	0.35	0.09	0.00	0.09	0.35	1.14
	4.9	0.20	0.31	0.10	0.41	0.07	0.00	0.07	0.32	1.11
	6.9	0.23	0.35	0.10	0.46	0.14	0.00	0.15	0.16	1.11
	9.0	0.31	0.38	0.07	0.45	0.01	0.10	0.11	0.13	1.03
	0.1	0.39	0.21	0.07	0.28	0.25	0.01	0.26	0.07	1.08
	4.4	0.19	0.29	0.04	0.34	0.08	0.02	0.10	0.38	1.15
0.7	4.9	0.20	0.30	0.10	0.41	0.07	0.00	0.07	0.32	1.11
	6.9	0.23	0.30	0.10	0.40	0.14	0.03	0.17	0.20	1.11
	9.0	0.33	0.31	0.12	0.43	0.12	0.00	0.12	0.12	1.21
	0.1	0.28	0.24	0.07	0.31	0.33	0.00	0.33	0.08	1.04
	4.4	0.20	0.32	0.06	0.37	0.10	0.00	0.10	0.33	1.09
0.9	4.9	0.20	0.32	0.07	0.39	0.06	0.00	0.06	0.34	1.06
	6.9	0.26	0.31	0.09	0.39	0.11	0.01	0.12	0.23	1.02
	9.0	0.33	0.34	0.10	0.43	0.11	0.00	0.11	0.12	1.19
	0.1	0.31	0.19	0.13	0.33	0.18	0.11	0.29	0.08	1.08
	4.4	0.21	0.28	0.05	0.33	0.10	0.00	0.10	0.36	1.06
1.0	4.9	0.20	0.32	0.07	0.39	0.07	0.00	0.07	0.35	1.10
	6.9	0.27	0.32	0.09	0.41	0.12	0.00	0.12	0.20	1.23
	9.0	0.34	0.31	0.12	0.42	0.12	0.00	0.12	0.11	1.23
	0.1	0.39	0.21	0.08	0.29	0.20	0.04	0.24	0.08	1.11
	4.4	0.19	0.33	0.05	0.38	0.07	0.02	0.10	0.33	1.08
2.0	4.9	0.21	0.24	0.07	0.31	0.11	0.01	0.13	0.35	1.13
	6.9	0.28	0.29	0.09	0.39	0.16	0.00	0.17	0.16	1.19
	9.0	0.30	0.29	0.12	0.41	0.17	0.00	0.18	0.11	1.02
	0.1	0.40	0.21	0.09	0.29	0.15	0.07	0.23	0.08	1.17
	4.4	0.20	0.36	0.04	0.40	0.10	0.01	0.11	0.29	1.11
3.0	4.9	0.20	0.29	0.05	0.35	0.08	0.00	0.08	0.37	1.09
	6.9	0.26	0.28	0.12	0.40	0.08	0.05	0.13	0.20	1.10
	9.0	0.32	0.27	0.12	0.39	0.17	0.01	0.18	0.11	1.04
	0.1	0.38	0.20	0.08	0.28	0.19	0.10	0.29	0.04	1.19
	4.4	0.19	0.41	0.06	0.47	0.09	0.02	0.11	0.22	1.07
4.0	4.9	0.18	0.26	0.02	0.28	0.14	0.00	0.14	0.40	1.11
	6.9	0.22	0.26	0.07	0.33	0.26	0.01	0.27	0.17	1.14
	9.0	0.28	0.29	0.12	0.40	0.19	0.02	0.21	0.11	1.08
	0.1	0.34	0.28	0.09	0.36	0.21	0.06	0.26	0.04	1.06
	4.4	0.18	0.48	0.06	0.54	0.11	0.01	0.12	0.16	1.06
5.0	4.9	0.19	0.25	0.04	0.29	0.10	0.01	0.11	0.40	1.27
	6.9	0.19	0.27	0.09	0.36	0.11	0.06	0.17	0.27	1.09
	9.0	0.28	0.25	0.03	0.27	0.28	0.00	0.28	0.16	1.14

Table S4. Parameters retrieved using the FBM analysis of the monomer decays of Py(x)-P*DL*GA in DMF solutions containing GdHCl.

[GdHCl] (M)	τ_M (ns)	k_2 (ns^{-1})	x (mol%)	f_{Mk2}	k_e(blob) (μs^{-1})	<n>	k_{blob} (μs^{-1})	f_{Mdiff}	f_{Mfree}	χ^2
			6.0	0.14	0.2	0.81	12.5	0.50	0.36	1.19
			8.0	0.29	3.8	1.41	14.7	0.39	0.32	1.16
0.0	215	0.147	10.4	0.26	2.7	1.51	12.0	0.45	0.29	1.21
			11.5	0.22	3.4	1.34	13.9	0.50	0.28	1.29
			12.4	0.25	3.0	1.50	12.9	0.50	0.24	1.13

0.7	205	0.085	6.0	0.20	0.0	0.88	10.6	0.44	0.36	1.09
			8.0	0.26	0.7	1.28	10.1	0.43	0.31	1.02
			10.4	0.36	0.4	1.46	8.9	0.45	0.19	1.17
			11.5	0.30	4.2	1.84	8.4	0.49	0.22	1.09
			12.4	0.30	3.9	1.65	9.2	0.47	0.23	1.07
1.0	204	0.077	6.0	0.22	0.0	0.80	10.6	0.45	0.33	1.13
			8.0	0.27	0.5	1.04	10.1	0.49	0.24	1.29
			10.4	0.38	0.4	1.41	8.4	0.44	0.18	1.26
			11.5	0.32	4.7	1.67	9.3	0.48	0.20	1.09
			12.4	0.38	5.0	1.79	9.3	0.45	0.17	1.07
3.0	197	0.052	6.0	0.28	2.4	0.84	5.7	0.40	0.32	1.11
			8.0	0.30	1.2	1.34	6.4	0.39	0.31	1.12
			10.4	0.35	0.7	1.60	5.5	0.42	0.22	1.22
			11.5	0.35	3.3	1.71	5.1	0.44	0.21	1.12
			12.4	0.34	3.3	1.75	6.0	0.47	0.19	1.11
5.0	191	0.040	6.0	0.19	0.0	0.95	5.9	0.33	0.48	1.00
			8.0	0.27	0.6	0.96	5.6	0.45	0.27	1.16
			10.4	0.30	2.1	1.43	5.3	0.41	0.29	1.09
			11.5	0.28	3.4	1.40	4.7	0.46	0.26	1.10
			12.4	0.31	2.7	1.72	4.1	0.47	0.22	1.09

Table S5. Parameters retrieved using the FBM analysis of the excimer decays of Py(*x*)-P*DL*GA in DMF solutions containing GdHCl.

[GdHCl] (M)	k_2 (ns^{-1})	x (mol%)	f_{Ek2}	τ_{E0} (ns)	$f_{EdiffE0}$	f_{EE0}	τ_D (ns)	f_{EdiffD}	f_{ED}	χ^2
0.0	0.147	6.0	0.20	38	0.64	0.05	105	0.10	0.00	1.19
		8.0	0.40	47	0.37	0.06	77	0.17	0.00	1.16
		10.4	0.35	42	0.41	0.07	75	0.18	0.00	1.21
		11.5	0.27	44	0.43	0.12	77	0.19	0.00	1.29
		12.4	0.30	42	0.40	0.11	74	0.19	0.01	1.13
0.7	0.085	6.0	0.28	48	0.52	0.12	123	0.08	0.00	1.09
		8.0	0.32	44	0.42	0.14	87	0.12	0.00	1.02
		10.4	0.38	46	0.38	0.14	85	0.10	0.00	1.17
		11.5	0.31	46	0.39	0.17	81	0.12	0.00	1.09
		12.4	0.34	45	0.38	0.13	77	0.16	0.00	1.07
1.0	0.077	6.0	0.29	49	0.52	0.12	123	0.08	0.00	1.13
		8.0	0.32	45	0.44	0.12	93	0.12	0.00	1.29
		10.4	0.40	48	0.37	0.14	86	0.09	0.00	1.26
		11.5	0.34	47	0.37	0.17	77	0.13	0.00	1.09
		12.4	0.37	48	0.33	0.19	74	0.11	0.00	1.07
3.0	0.052	6.0	0.35	59	0.29	0.09	66	0.20	0.07	1.11
		8.0	0.37	50	0.37	0.16	93	0.10	0.00	1.12
		10.4	0.39	47	0.36	0.15	82	0.11	0.00	1.22
		11.5	0.38	48	0.34	0.14	77	0.13	0.00	1.12
		12.4	0.35	45	0.37	0.17	76	0.12	0.00	1.11
5.0	0.040	6.0	0.29	54	0.46	0.18	139	0.06	0.02	1.00
		8.0	0.32	50	0.45	0.13	105	0.09	0.00	1.16
		10.4	0.35	49	0.38	0.16	93	0.10	0.00	1.09
		11.5	0.33	49	0.42	0.13	90	0.11	0.00	1.10

	12.4	0.35	46	0.39	0.13	80	0.13	0.00	1.09

Table S6. Global fractions of pyrene species calculated using parameters retrieved from the FBM analysis of Py(x)-P*DL*GA in DMF solutions containing GdHCl.

[GdHCl] (M)	x (mol%)	f_{k2}	f_{diffE0}	f_{diffD}	f_{diff}	f_{E0}	f_D	f_{agg}	f_{free}	χ^2
0.0	6.0	0.13	0.42	0.07	0.49	0.03	0.00	0.03	0.35	1.19
	8.0	0.28	0.26	0.12	0.37	0.04	0.00	0.04	0.30	1.16
	10.4	0.25	0.30	0.13	0.43	0.05	0.00	0.05	0.27	1.21
	11.5	0.20	0.32	0.14	0.46	0.09	0.00	0.09	0.26	1.29
	12.4	0.23	0.31	0.15	0.46	0.08	0.01	0.09	0.22	1.13
0.7	6.0	0.19	0.35	0.05	0.40	0.08	0.00	0.08	0.33	1.09
	8.0	0.23	0.30	0.09	0.39	0.10	0.00	0.10	0.28	1.02
	10.4	0.32	0.32	0.08	0.40	0.12	0.00	0.12	0.17	1.17
	11.5	0.26	0.32	0.10	0.42	0.14	0.00	0.14	0.19	1.09
	12.4	0.27	0.30	0.12	0.42	0.10	0.00	0.10	0.21	1.07
1.0	6.0	0.20	0.36	0.05	0.42	0.08	0.00	0.08	0.30	1.13
	8.0	0.25	0.35	0.09	0.44	0.10	0.00	0.10	0.22	1.29
	10.4	0.34	0.31	0.07	0.39	0.12	0.00	0.12	0.16	1.26
	11.5	0.28	0.31	0.10	0.41	0.14	0.00	0.14	0.17	1.09
	12.4	0.32	0.28	0.09	0.38	0.17	0.00	0.17	0.14	1.07
3.0	6.0	0.25	0.21	0.15	0.35	0.07	0.05	0.12	0.28	1.11
	8.0	0.27	0.27	0.07	0.34	0.12	0.00	0.12	0.28	1.12
	10.4	0.31	0.29	0.09	0.37	0.12	0.00	0.12	0.20	1.22
	11.5	0.31	0.28	0.11	0.39	0.11	0.00	0.11	0.18	1.12
	12.4	0.29	0.31	0.10	0.40	0.14	0.00	0.14	0.16	1.11
5.0	6.0	0.16	0.26	0.03	0.30	0.10	0.01	0.11	0.43	1.00
	8.0	0.24	0.34	0.07	0.41	0.10	0.00	0.10	0.25	1.16
	10.4	0.26	0.29	0.08	0.36	0.12	0.00	0.12	0.25	1.09
	11.5	0.26	0.32	0.09	0.41	0.10	0.00	0.10	0.23	1.10
	12.4	0.28	0.32	0.10	0.42	0.11	0.00	0.11	0.20	1.09
